# Sult2b1 deficiency exacerbates ischemic stroke by promoting pro-inflammatory macrophage polarization in mice

**DOI:** 10.7150/thno.61646

**Published:** 2021-11-01

**Authors:** Yan Wang, Haojie Jin, Yafang Wang, Yang Yao, Cuixia Yang, Jihong Meng, Xiaomu Tan, Yu Nie, Lixiang Xue, Baohui Xu, Heng Zhao, Feng Wang

**Affiliations:** 1 Department of Neurosurgery, Stanford University School of Medicine, Stanford, USA.; 2 Institute of Medical Innovation and Research, Peking University Third Hospital, Beijing, China.; 3 Medical Research Center, Peking University Third Hospital, Beijing, China.; 4 The College of Forestry, Beijing Forestry University, Beijing, China.; 5 Department of Plant Biology, Carnegie Institution for Science, Stanford, USA.; 6 Shanghai Institute of Immunology, Translational Medicine Center, Shanghai General Hospital, Shanghai Jiao Tong University School of Medicine, Shanghai, China.; 7 Research Center of Translational Medicine, Shanghai Children's hospital, State Key Laboratory of Oncogenes and Related Genes, Shanghai Jiao Tong University School of Medicine, Shanghai, China.; 8 Fuwai Hospital, National Centre for Cardiovascular Diseases, Chinese Academy of Medical Sciences, Beijing, China.; 9 Division of Vascular Surgery, Department of Surgery, Stanford University School of Medicine, 1201Welch Road, MSLS Building, Stanford, USA.

**Keywords:** Ischemic Stroke, Sult2b1, Cholesterol Sulfate, Macrophage, Neuroinflammation

## Abstract

**Rationale:** Stroke is a leading causes of human death worldwide. Ischemic damage induces the sterile neuroinflammation, which directly determines the recovery of patients. Lipids, a major component of the brain, significantly altered after stroke. Cholesterol sulfate, a naturally occurring analog of cholesterol, can directly regulate immune cell activation, indicating the possible involvement of cholesterol metabolites in neuroinflammation. Sulfotransferase family 2b member 1 (Sult2b1) is the key enzyme that catalyzes the synthesis of cholesterol sulfate. This study aimed to investigate the function of Sult2b1 and cholesterol sulfate in the neuroinflammation after ischemic stroke.

**Methods and Results**: *Sult2b1*^-/-^ and wild-type mice were subjected to transient middle cerebral artery occlusion. Our data showed that *Sult2b1*^-/-^ mice had larger infarction and worse neurological scores. To determine whether immune cells were involved in the worsening stroke outcome in *Sult2b1*^-/-^ mice, bone marrow transplantation, immune cell depletion, and adoptive monocyte transfer were performed. Combined with CyTOF and immunofluorescence techniques, we demonstrated that after stroke, the peripheral monocyte-derived macrophages were the dominant cell type promoting the pro-inflammatory status in *Sult2b1*^-/-^mice. Using primary bone marrow-derived macrophages, we showed that cholesterol sulfate could attenuate the pro-inflammatory polarization of macrophages under both normal and oxygen-glucose deprivation conditions by regulating the levels of nicotinamide adenine dinucleotide phosphate (NADPH), reactive oxygen species (ROS), and activating the AMP-activated protein kinase (AMPK) - cAMP responsive element-binding protein (CREB) signaling pathway.

**Conclusions:** *Sult2b1*^-/-^ promoted the polarization of macrophages into pro-inflammatory status. This trend could be attenuated by adding cholesterol sulfate, which promotes the polarization of macrophages into anti-inflammatory status by metabolic regulation. In this study, we established an inflammation-metabolism axis during the macrophage polarization after ischemic stroke.

## Introduction

Ischemic stroke is the third leading mortality worldwide 1, 2. Stroke injury reduces glucose and oxygen supply, releases damage-associated molecular patterns and causes neural cell damage by secreting pro-inflammatory cytokines and chemokines 3, 4. Furthermore, the peripheral immune cells are recruited from blood vessels into the ischemic hemisphere and further aggravate neuroinflammation within minutes or even prolonged in days and weeks 5. Therefore, understanding the immune response induced by ischemic stroke will help to establish the therapeutic strategies for clinical patient treatment.

Sulfotransferase family 2b member 1 (Sult2b1) is the key enzyme that catalyzes cholesterol synthesized to cholesterol sulfate. As one of the most important known sterol sulfates in human plasma, it is presented as a normal constituent in various human tissues, including the brain. Cholesterol sulfate has also been reported play curtail roles in regulating immune response. We previously showed that cholesterol sulfates abolished T cell activation by inhibiting the CD3 ITAM phosphorylation 6. Furthermore, mice lacking sulfotransferase increased the infiltration of immune cells in the eye 7.

The brain is a lipid-rich tissue, and almost 60% of the brain is composed of fat 8. These lipids are required to maintain the brain structure and function 9. As early as in 1976, cholesterol sulfate was shown closely related to rat brain development 10. Recently, cholesterol sulfate has been further shown had protective role against oxidative stress in brain astrocyte 11. In addition, abnormal lipid expression is closely associated with various brain diseases 9. For instance, the up-regulated cholesterol level increases risk of Alzheimer's Disease 12. The transportation deficiency of cholesterol has been seen in Niemann-pick disease 13. In ischemic stroke, the level of activated phospholipase was increased, whereas that of docosahexaenoic acid (DHA) decreased 14, and the level of total cholesterol was positively associated with ischemic stroke occurrences 15, 16. However, the function of cholesterol sulfate in ischemic stroke, especially in ischemia induced neuroinflammation is not clear.

In present study, we investigated the significance and potential mechanisms of *Sult2b1* in ischemic stroke. We found that the after ischemia, *Sult2b1* deficiency mice had enlarged infarction size and worsened neurological behaviors in association with augmented monocyte migration to inflamed brain and pro-inflammatory macrophage polarization. In macrophages, exogenous cholesterol sulfates increased the level of NADPH but reduced that of ROS. The AMPK-CREB signaling pathway was considered as the potential mechanism as the total expression and phosphorylation of AMPK and CREB were both increased when treated with cholesterol sulfate. This study established an inflammation-metabolism axis of the ischemic stroke through the polarization of macrophages.

## Materials & Methods

### Animals

As we described in a previous study 6, *Sult2b1^-/-^* mice were backcrossed onto the C57BL/6 genetic background and confirmed by genotyping. C57BL/6 mice were purchased from Jackson Laboratory and Department of Laboratory Animal Science, Peking University. Five mice were housed in each cage under a 12:12 h light-dark cycle (lights on at 07:00 am), at a temperature of 25 - 27 °C and 40 - 60 % humidity, with freely available food and water at Stanford University and Peking University. All animal experiments were performed under the protocols based on the Animal Research Reporting *In vivo* Experiments Guidelines 17, and approved by the Institutional Animal Care and Use Committee. Animals were randomly assigned to groups; behavior tests and treatments were conducted in a blinded fashion - the researchers performing the surgeries were blinded to the animal groups. A total of 213 male mice were included in this study. Animals included into infarction and behavioral test were based on the following exclusion criteria: (1) had no neurological deficits after stroke; (2) brains had evidence of surgical subarachnoid hemorrhage (no more than two mice were excluded from each group); and (3) did not fit the baseline of the behavioral test before surgery for that test.

### Focal cerebral ischemia

Anesthesia was induced with 5% isoflurane and maintained at 1-2% isoflurane throughout the surgery. Body temperature was maintained at 37 ± 0.5 °C with a surface heating pad during the entire procedure. Focal cerebral ischemia was induced by 45 min of transient middle cerebral artery occlusion (MCAo) by inserting a silicone-coated 6-0 monofilament (Doccol Corp, CA, USA) into the left common carotid artery to block the MCA, as previously reported 18. Sham-operated mice underwent the same procedure, but without monofilament insertion.

### Infarction measurement

At 72 h after ischemia, the mice were deeply anesthetized with isoflurane and euthanized. The brains were sliced into five slices of 2 mm thickness and stained in 2% 2, 3, 5-triphenyltetrazolium chloride (TTC staining, Cat^#^ T8877, Sigma Aldrich, MO, USA) for 10 - 15 min at 37 °C and fixed in 4% paraformaldehyde (PFA) overnight. Brain infarctions were then measured using ImageJ software 19. The images were normalized to the contralateral hemisphere and expressed as a ratio according to the following formula: (area of the non-ischemic hemisphere - area of the non-ischemic tissue in the ischemic hemisphere) / area of non-ischemic hemisphere 20.

### Neurobehavioral examination

Neurobehavioral tests were conducted by an investigator who was blinded to the treatment. This assay is based on a modified neurological severity score (mNSS) system to present a comprehensive assessment of neurological function including motor, sensory, balance, and reflex tests 21. The mNSS scores range from 0 to 14, where 0 represents normal and 14 represents the highest degree of neurological deficiency. For the motor assay, after raising the mouse by the tail, the bending and torsion of the limbs were observed (score 0-3). Walking posture was also checked (score 0-3). For the balance test, mice were placed on a beam to evaluate whether they could maintain their balance and whether their limbs fell off the beam or they could walk on the beam (score 0-6). For the sensory and reflex tests, the pinna and corneal reflexes were examined, respectively (score 0-2). The rotating beam test was used to evaluate neurological deficits in coordination and integration of movement in mice after ischemic stroke. The mice were trained to walk along a 100-cm-long rotating wooden beam (80 mm in diameter, approximately 80 cm above the floor, at 3 rpm rotation) before the surgery. Only the mice that passed the initial baseline test were included in the following behavioral test experiment.

### Cerebral blood flow (CBF) monitor

As we previously described, a laser Doppler probe was used to test CBF 22. Briefly, 1 mm diameter holes were drilled to monitor CBF at the ischemic core (1.0 mm posterior to the bregma, 4.5 mm lateral to the midline) and ischemic margin (2 mm posterior to the bregma, 2 mm lateral to the midline) in animals subjected to suture middle cerebral occlusion. To attach the Doppler probe to the skull table, a sagittal cut of the scalp was performed, with the probe placed 4 mm lateral and 1 mm posterior to the bregma, fixed in place with glue. CBF was measured 15 min before ischemia onset (baseline), and during ischemia. Subjects in whom blood flow did not remain below 30% of baseline values for the entire period were excluded from the analysis. The blood flow of the ischemic and non-ischemic hemispheres was monitored during the surgery to ensure that the surgery was successfully performed for all of the mice used in the behavioral tests (Table S1).

### Real-time PCR

The RNA from the ischemic hemisphere and the peripheral blood mononuclear cells (PBMCs) was purified using the RNeasy Mini Kit (Cat^#^ 74104, Qiagen, Valencia, USA). The RNA from BMDMs was purified using RNA simple total RNA kit (Cat^#^ DP419, TIANGEN Biotech, Beijing, China). The RNA quality was measured using a Bioanalyzer. GoScript^TM^ Reverse transcription system was used for reverse transcription (Cat^#^ A5000, Promega, USA) and the primer sequences are listed in Table S2. The primers to detect *Sult2b1, Sts, Papss1, and Papss2* were performed using commercial TaqMan systems. *Sult2b1* TaqMan primer (Assay ID: Mm00450550), *Sts* (Assay ID: Mm04214605), *Papss1* (Assay ID: Mm00442283), *Papss2* (Assay ID: Mm01197820), and reference gene *β-actin* (Assay ID: Mm00615581_s1) were purchased from Thermo Fisher Scientific (Waltham, USA).

### Cholesterol sulfate level detection

As described in our previous study 6, cholesterol-26,26,26,27,27,27-d6 and 5,24-cholestadien-3β-ol sulfate sodium salt were added to the extraction as internal controls for cholesterol and cholesterol sulfate, respectively. The level of cholesterol sulfate was measured at the Stanford University Mass Spectrometry facility.

### Luminex assay

The cell supernatant was subjected to a hard spin (10,000 g) before freezing to remove cells and debris that could clog the Luminex instrument. Tissue homogenization from the ischemic hemisphere was collected using tissue extraction reagent 1 (Cat^#^ FNN0071, Thermo Fisher, USA), with protease and phosphatase inhibitor (Cat^#^ 78441, Halt^TM^ protease and phosphatase inhibitor cocktail, Thermo Fisher, USA). Luminex assays were performed at the Human Immune Monitoring Center at Stanford using a mouse 39-plex panel.

### Cell migration assay

BMDMs were treated with two CXCL1 inhibitors, SB225002 (Cat^#^ s7651, Selleck, China) and SB265610 (Cat^#^17879, Cayman, USA), separately. DMSO was served as a control group. The treated cells were plated in a 24-well transwell plate (Cat^#^ 3492, Transwell® Inserts, Sterile, Corning®, MA, USA). The chemokines CCL2 (Cat^#^ 578402, MCP1, BioLegend®, CA, USA) was added into the lower chamber at 2 ng/mL 23. After 4 h of incubation at 5% CO2, and 37 °C, cells from the upper and lower chambers were collected and counted.

### Bone marrow transplantation

B6.SJL-Ptprca Pepcb/BoyJ (CD45.2^-^) mice (stock number 002014) were purchased from Jackson Laboratory. The details of bone marrow transplantation are the same as we previously described 6, 24. Briefly, male WT B6.SJL mice were subjected to irradiation (950 rad), and 5 × 10^6^ bone marrow cells from WT C57BL/6J (CD45.2^+^) or *Sult2b1^-/-^* (CD45.2^+^) mice were i.v. injected into each irradiated recipient. Eight weeks after bone marrow transfer, peripheral blood CD45.2 expression was detected by flow cytometry (FACS) to ensure the successful bone marrow transfer. For the bone marrow chimera experiment, B6.SJL-Ptprca Pepcb/BoyJ mice were used. Male WT B6.SJL mice were subjected to lethal irradiation (950 rad) and then reconstituted with 1:1 mixed bone marrow of WT B6.SJL (CD45.2^-^) and WT B6 (CD45.2^+^) or WT B6.SJL (CD45.2^-^) and *Sult2b1^-/-^* (CD45.2^+^) mice. Bone marrow cells (5 × 10^6^) were i.v. injected into each irradiated recipient.

### Monocyte and neutrophil depletion

For the monocyte depletion experiment, mice was i.v. injected clodronate liposome (SKU: CP-005-005, Liposoma, Netherland) 1 day before, and 1 day after surgery. For Neutrophil depletion, mice was i.p. injected the Mab MAB anti-mouse Ly6G antibody (clone 1A8) (Bio X Cell, W., NH, USA) 1 day before and 1 day after surgery (250 µg / mouse).

### Monocyte adoptive transfer

B6.SJL-Ptprca Pepcb/BoyJ (CD45.2^-^) mice were used as the recipient mice for the adoptive monocyte transfer experiments. Monocytes were first depleted using clodronate liposome via i.v. injection. After 36 h, purified monocytes from *Sult2b1^-/-^* mice (CD45.2^+^) or WT C57BL/6 mice (*Sult2b1^+/+^*, CD45.2^+^) were adoptively transferred via i.v. injection. The CD45.2 expression was monitored by FACS.

### Primary monocyte isolation, polarization

Primary bone marrow monocytes from C57BL/6 or *Sult2b1^-/-^* mice were first treated with red blood cell lysis buffer (Cat^#^ 00-4300-54, eBioscience, CA, USA). Then, the cells were plated in 24-well plates at 37 °C in a 5% CO2 incubator. After 12 h, cell media was exchanged with 10 ng/mL Macrophage colony stimulation factor (M-CSF) recombinant mouse protein (Cat^#^ PMC2044, Thermo Fisher Scientific, Waltham, MA, USA) for 6 days, which differentiated peripheral monocytes into the neutral state macrophages, M (M-CSF). M (M-CSF) can be further polarized to the pro-inflammatory macrophages, M (LPS) using 1 mg/mL lipopolysaccharides (Cat^#^ L2880, LPS; Sigma-Aldrich, St. Louis, MI, USA), or anti-inflammatory macrophages, M(IL-4) using 2 mg/mL recombinant murine IL-4 (Cat^#^ 214-14, PeproTech Sciences, Inc., Ontario, Canada). Cholesterol sulfate (Cat^#^ 700016P-25MG, Sigma, USA) were added to the cell culture at the indicated time points. For oxygen-glucose deprivation (OGD) model, BMDMs were transferred to deoxygenated, glucose-free DMEM cell culture media. Then, cells were placed in an oxygen deprivation box and incubated with 1% O_2_, 5% CO_2_, and 95% N_2_ at 37 °C for 4-6 h to establish the OGD injury model. Following OGD, the cells were transferred to normal cell culture media and cultured with 5% CO_2_ at 37 °C for overnight for the re-oxygen and re-glucose treatment.

### CyTOF

Cell isolation procedures were performed on ice, as we previously published 25. The ischemic hemisphere was minced in RPMI 1640 and filtered through a 70 µm cell strainer. Two milliliters of 70% Percoll were loaded to the bottom of the cell suspension, centrifuged at 600 g for 30 min. The interphase cells were collected and re-suspended in PBS. Cell pellets were stained in a 100 µL solution of Cell-ID Cisplatin (Fluidigm, CA, USA) for live and dead staining. After 5 min incubation, the staining was quenched with 500 µL Maxpar cell staining buffer (Fluidigm, CA, USA). Samples were next barcoded using a 20-Plex Pd barcoding system and re-suspended in 50 µL Maxpar cell staining buffer. Fc-Receptor blocking solution (BioLegend, CA, USA) was added and incubated for 10 min, and 50 µL of antibody cocktail was added to staining cell markers. A list of antibodies is summarized in Table S3. Samples were injected into the CyTOF machine (CyTOF2, Fluidigm, CA, USA) at Stanford FACS facility.

### Immunofluorescence staining

Brains were collected after PBS perfusion and immediately frozen, then cut into 20 μm coronal sections on a cryostat (Leica, Wetzlar, Germany). Sections were collected on premium charged microscope slides, and stored at -80 °C. Sections were first treated with 4% PFA for 30 min at 37 °C, washed with PBS twice, each for 15 min, and treated with 0.3% triton-100 in PBS for 30 min and blocked with 10% BSA for 1 h. Primary antibodies were added to each slide at a 1:100 dilution at 4 °C overnight. Anti-mouse CD68 antibody (Cat^#^ ab955, Abcam, USA), rabbit anti-mouse iNOS (Cat^#^ ab15323, Abcam, USA) were used as primary antibodies. After three washes with PBS, the sections were incubated with the secondary antibodies (1:200), Alexa 488-conjugated goat anti-mouse antibody (Cat^#^ A-11001), Alexa 488-conjugated goat anti-rabbit antibody (Cat^#^ A11008) and Alexa 594-conjugated donkey anti-mouse antibody (Cat^#^ A21203) for 60 min at RT. All of the secondary antibodies were purchased from Invitrogen (USA). After washing with PBS, the stained sections were mounted with DAPI.

### ELISA

The cell supernatant was collected and centrifuged for 20 min at 1000 g at 4 °C. 100 µL of each dilution of samples were added into the appropriate wells. The plate was covered with a sealer and incubated for 90 min at 37 °C. The liquid was removed from each well, and 100 µL of biotinylated detection antibody working solution was added for 1 h at 37 °C. Then the plate was washed, and 100 µL of HRP-conjugated working solution was added for 30 min at 37 °C. After five washes, 90 µL of substrate reagent was added and incubated for 15 min at 37 °C. The stop solution was added within 30 min and the OD value was read at 450 nm (MouseIL-10 ELISA Kit, E-EL-M0046, Elabscience, China). The intracellular cAMP level was measured using a commercially available ELISA kit from Cloud-Clone Crop (Wuhan, China). Briefly, the cultured cells were washed using cold PBS and suspended in 1 ml ice-cold 65% ethanol. Then the cell lysates were centrifuged at 2,000 g for 10 min and the supernatants collected 26. The extracts were dried under vacuum and dissolved in 0.5 ml assay buffer, and the intracellular cAMP levels were detected following the manufacturer's protocol.

### NADPH quantification assay

The primary BMDMs were plated in six-well plates at 1×10^6^ cell / well. Cells were treated under normal conditions or OGD conditions, with or without cholesterol sulfate for the designated time. Then, 200 µL NADP^+^/ NADPH extraction solution (Cat^#^ S0179, Beyotime, China) and the extracts were centrifuged at 12000 g for 8 min at 4 °C to collect the supernatant. The supernatant (50 µL) was added to the 96 well plates together with G6PDH working solution and incubated at 37 °C for 10 min. Then the absorbance was read at 450 nm absorbance.

### Mitochondrial ROS assay

The MitoSOX^TM^ red mitochondrial superoxide indicator* for live cells (Cat^#^ MP36008, Invitrogen, USA), which is a fluorogenic dye for highly selective detection of superoxide in the mitochondria of live cells, was used in this experiment. As indicated in the product details, the MitoSOX^TM^ was first diluted into a working solution, then applied 1.0-2.0 mL of MitoSOX^TM^ reagent in each sample. The data were collected using a florescent microscopy.

### Western blotting

Cell samples were collected in RIPA buffer after washing with cold PBS. Primary antibodies, anti-AMPKα mAb (Cat^#^2793, Cell Signaling, USA), Phospho-AMPKα mAb (Cat^#^2535, Cell Signaling, USA), iNOS mAb (MAB9502-SP, R&D systems, USA), anti-CREB mAb (Cat^#^9197, Cell Signaling, USA), anti-phospho-CREB(ser133) mAb (Cat^#^9198, Cell Signaling, USA) were used at 1:1000 dilution. Anti-mouse HRP was used as a secondary antibody at 1:2000 dilution (Cat^#^ ZB-2305, Zsbio, China). Anti-GAPDH (C1312, Applygen, China), anti-tubulin (Cat^#^ bsm-33034m, Bioss, China) were served as a loading controls.

### Statistical analysis

Animal numbers were calculated before to the experiments using G*Power software 27. For the comparison of the differences between two independent means with two tails, analysis was performed a priori with a given α (0.05), power (0.95). Significant differences among groups was determined by two - tailed unpaired and paired Student's *t*-test. For experiments with one treatment and more than two groups, a one-way analysis of variance (ANOVA) was applied. For experiments with more than one treatment or variants, two-way ANOVA was performed, followed by Tukey's multiple comparison test using GraphPad Prism 7.0 (GraphPad Software, CA, USA).

## Results

### *Sult2b1^-/-^* mice has a larger infraction and worse neurological phenotype than WT mice

**Figure 1:**
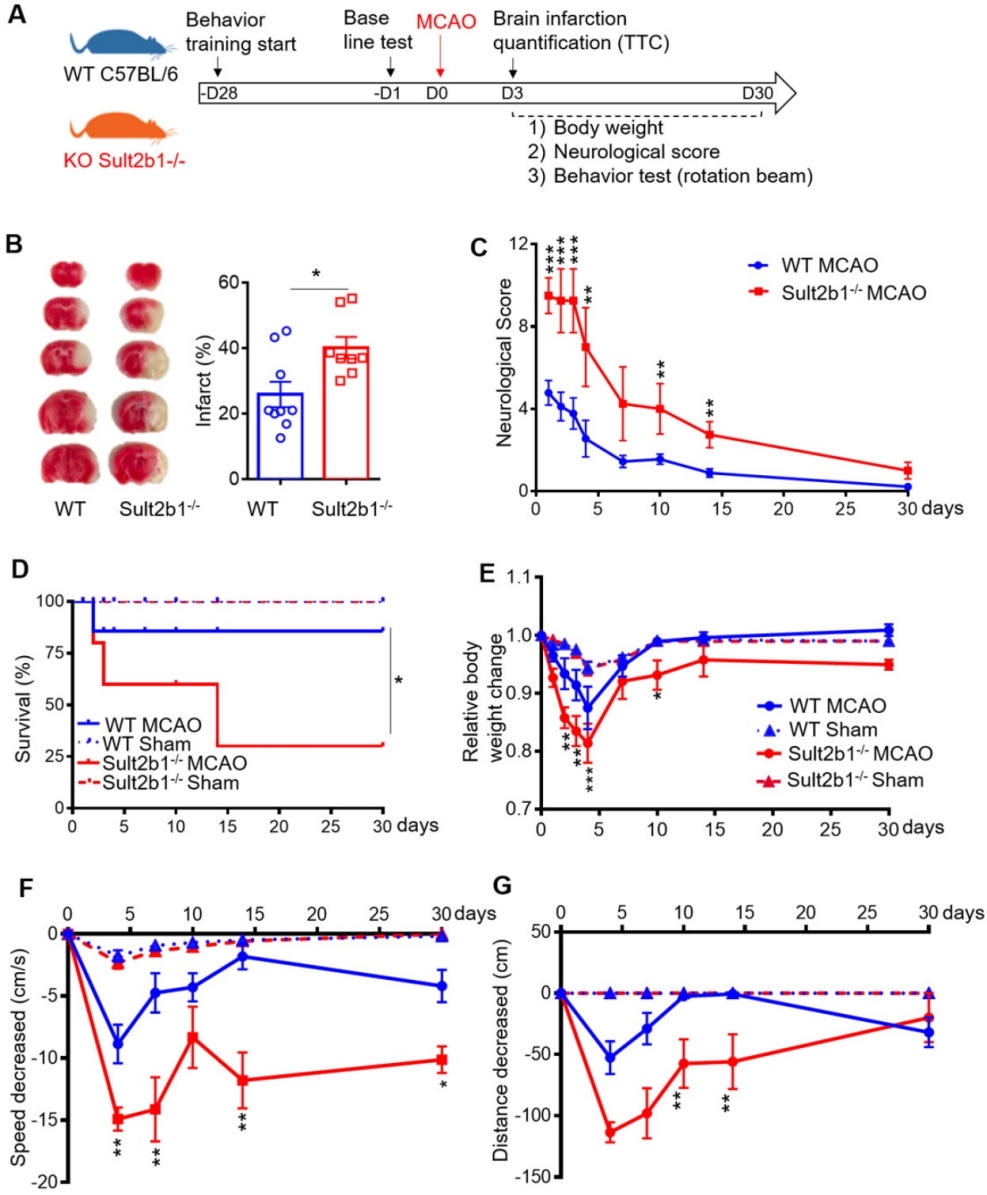
***Sult2b1^-/-^* mice exacerbates ischemic lesion size and behavioral outcome. (A)** Experiment design and timeline. Behavior training was started 4 weeks before the surgery. Baseline information was collected 1 day before surgery. Staining with 2, 3, 5-triphenyltetrazolium chloride (TTC) was conducted 3 days after stroke onset. Behavioral tests were performed on days 4, 7, 10, 14 and 30. **(B)** Infarction size at 3 days after stroke. Wild-type (WT) mice, n = 9; *Sult2b1^-/-^* mice, n = 8. **(C)** Neurological score data were obtained on days 1, 2, 3, 4, 7, 10, 14, and 30, based on the modified Neurological Severity Scores (mNSS). WT MCAo mice, n = 11; *Sult2b1^-/-^* MCAo mice, n = 11. **(D)** The death rate after ischemic stroke. WT MACo mice, n = 11; *Sult2b1^-/-^* ^/-^ MACo mice, n = 11; WT sham mice, n = 6; *Sult2b1^-/-^* sham mice, n = 5. **(E)** *Sult2b1^-/-^* mice had less body weight than WT mice. After stroke, the relative body weight change (= weight change / original body weight) in *Sult2b1^-/-^* mice decreased significantly compared to that in WT mice. **(F)** Speed changes in *Sult2b1^-/-^* mice were more significant than those in WT mice. **(G)** Decreased in distance covered by *Sult2b1^-/-^* mice were more significant than those in WT mice. * P < 0.05, **P < 0.01, ***P < 0.001 between *Sult2b1^-/-^* and WT mice. The blue solid lines with circles represent WT MCAo mice; the blue dash lines with triangles represent WT sham mice; the red solid lines with circles represent *Sult2b1^-/-^* MCAo mice, and the red dash lines with triangles represent *Sult2b1^-/-^* sham group. WT MACo mice, n = 11; *Sult2b1^-/-^* MACo mice, n = 11; WT sham mice, n = 6; *Sult2b1^-/-^* sham mice, n = 5 (E-G).

Both *Sult2b1^-/-^* mice and WT mice were subjected to MCAo (Figure 1A). TTC staining showed that *Sult2b1^-/-^* mice had larger infarction (Figure 1B) and worse neurological scores (Figure 1C) than WT mice. Furthermore, *Sult2b1^-/-^* mice had a lower survival rate than WT mice in 30 days after ischemic stroke (Figure 1D). The relative weight loss was more dramatic in *Sult2b1^-/-^* mice (Figure 1E). Furthermore, *Sult2b1^-/-^* mice had significant decrease in both speed and running distance, assayed by the previously reported rotation beam test 28 (Figure 1F-G). These results suggested that *Sult2b1* played an important role in determining stroke outcome.

### The level of cholesterol sulfate is down-regulated after ischemic stroke

**Figure 2:**
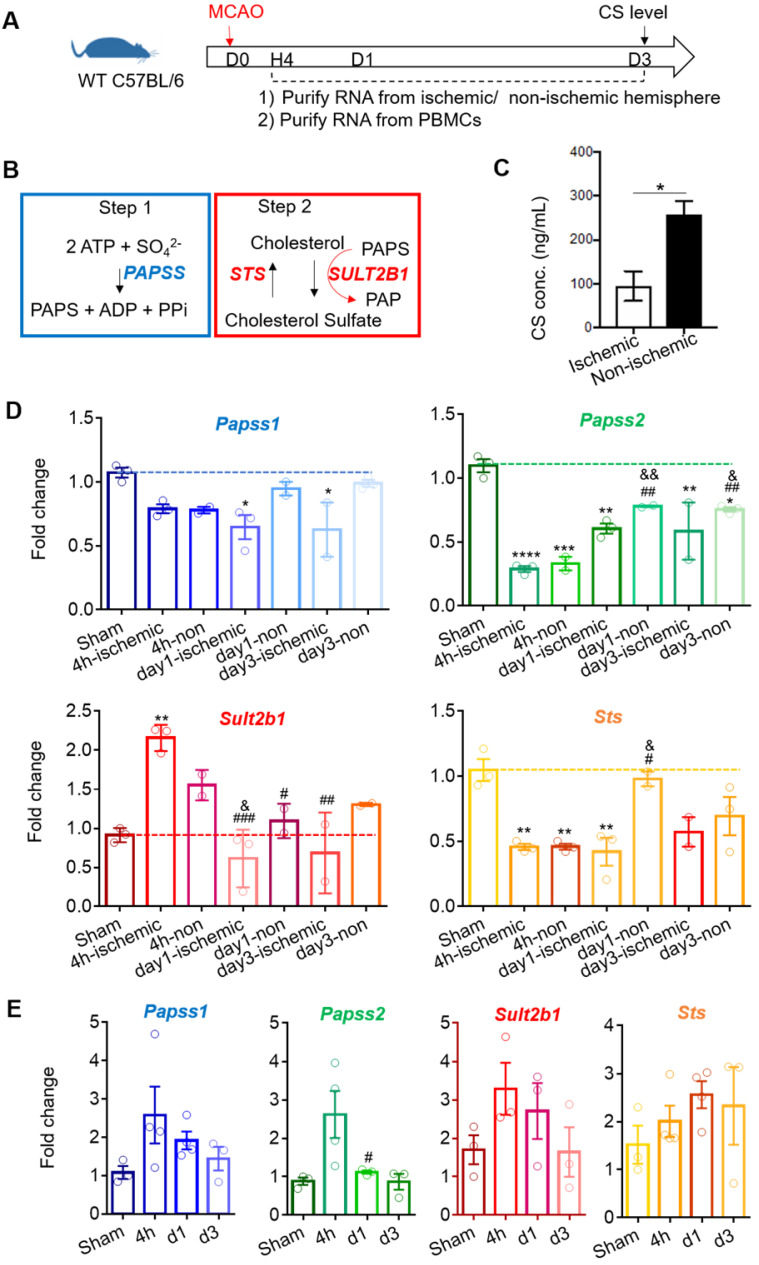
** Stroke induces *Sult2b1* and cholesterol sulfate expression in the ischemic hemisphere. (A)** The timeline of the experiment. RNAs from the ischemic/non-ischemic hemisphere and peripheral blood mononuclear cells (PBMCs) were collected at 4 h, 1 day, and 3 days after stroke onset. Three days after ischemic stroke, the brain ischemic/non-ischemic hemisphere was evaluated for cholesterol sulfate levels. **(B)** The two steps of cholesterol sulfate synthesis. **(C)** The level of cholesterol sulfate in the brain after ischemic stroke. **(D)** RT-PCR analyses of *Papss1*, *Papss2*, *Sult2b1,* and *Sts* in the ischemic and non-ischemic hemisphere from 4 h to day 7. * vs. sham, * P < 0.05, ** P < 0.01; # vs. 4h-ischemic hemisphere, # P < 0.05, ## P < 0.01; & vs. 4h-non-ischemic, & P < 0.05, && P < 0.01. **(E)** The RT-PCR analyses of *Papss1*, *Papss2*, *Sult2b1,* and *Sts* from PBMCs at various time points. # vs. 4h, # P < 0.05. n = 3. CS stands for cholesterol sulfate.

Using WT C57BL6 mice, we quantified the cholesterol sulfate level using mass spectrometry 3 days after stroke (Figure 2A). The cholesterol sulfate synthesis process consists of two steps: (1) the sulfate donor 3'-phosphoadenosine-5'-phosphosulfate (PAPS) is generated by PAPS synthase isoforms, PAPSS1 and PAPSS2, and (2) using PAPS as a substrate, Sult2b1 sulfates cholesterol to cholesterol sulfate (Figure 2B). The data showed that the cholesterol sulfate level in the ischemic hemisphere was remarkably lower than that in the non-ischemic hemisphere, suggesting that ischemic damage consumed a large amount of cholesterol sulfates, and the damaged area was still demanding for more cholesterol sulfate (Figure 2C). To further investigate the expression of the key molecules involved in the cholesterol sulfate biosynthesis process, we collected both brain mononuclear cells and PBMCs. After stroke, there was a dramatic decrease for both *Papss* isoforms in the ischemic hemisphere compared to sham control in the mouse brain. For *Sult2b1*, the expression in the ischemic hemisphere was significantly increased from 4 h after ischemia, while the *Sts* had decreased compared to the sham control groups (Figure 2D). In the peripheral blood, the relative expression changes of *Sult2b1* and *Sts* were not as dramatic as in the brain, but the expression of *Papss2* still significantly increased 4 h after ischemia (Figure 2E).

### Myeloid cells contributes to the enlarged infraction in *Sult2b1^-/-^* mice

**Figure 3:**
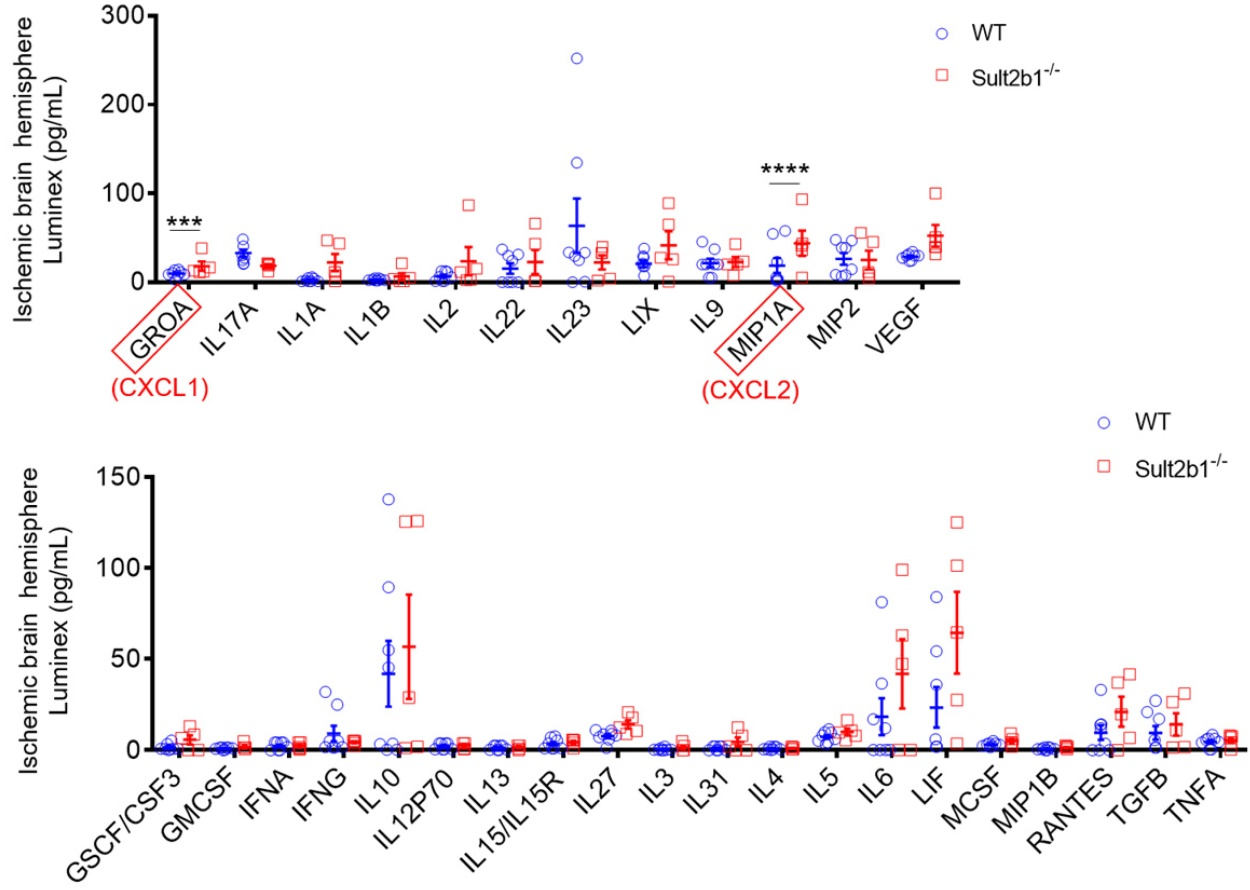
** Luminex assay of the ischemic hemisphere 3 days after stroke.** The protein lysates of ischemic hemispheres from WT and *Sult2b1^-/-^* mice were collected and tested using the Luminex assay. *** P < 0.001, **** P < 0.0001.

Next, we used a high-throughput Luminex assay to detect the cytokine and chemokine expression in the ischemic hemisphere 3 days after stroke. The data showed that two cytokines, GROA (CXCL1) and MIP1A (CXCL2) were dramatically increased in *Sult2b1^-/-^* mice (Figure 3). The CXCL1-CXCR2 axis has been reported to regulate the monocyte infiltration after cardiac hypertrophy and eventually heart failure 29, 30. Increased CXCL2 is also associated with increased neutrophil numbers 31. To confirm the crucial role of CXCL1, we preformed cell migration assay using transwell system. When CXCL1 inhibitor (SB265610 or SB225001) was added to the system, the migration capabilities of BMDMs were significantly decreased, compared to those treated with DMSO (Figure S1). These results led us to question whether the worse outcome of *Sult2b1^-/-^* mice was due to the myeloid cells.

**Figure 4:**
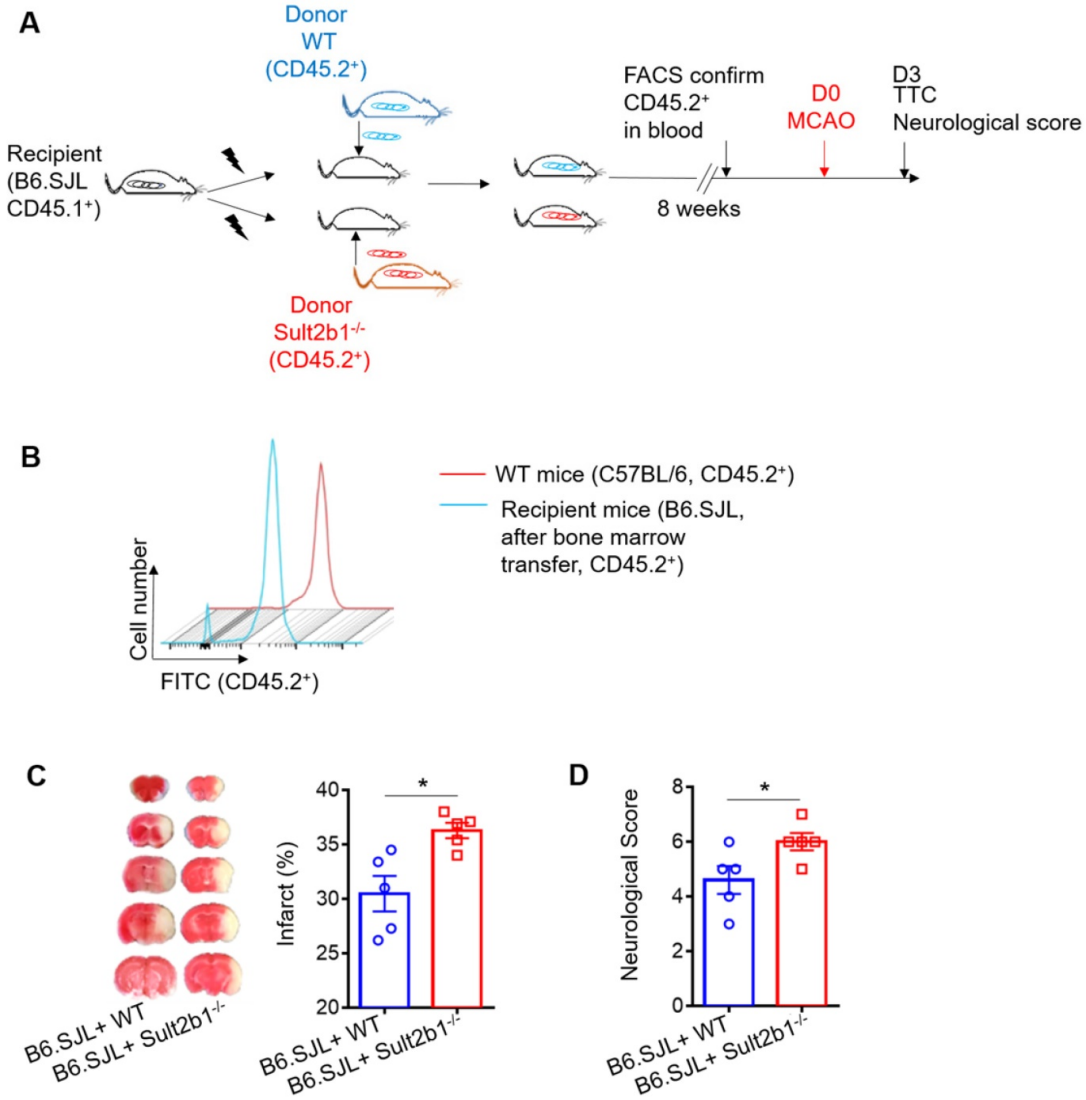
** Circulating leukocytes are critical for the protective effects of *Sult2b1^-/-^* on stroke. (A)** Experimental procedures to study the effect of bone marrow transfer on stroke outcomes: after irradiation, recipient WT mice (CD45.2^-^, *Sult2b1^+/+^*) received bone marrow cells from WT mice (CD45.2^+^, *Sult2b1^+/+^*) or *Sult2b1^-/-^* mice (CD45.2^+^, *Sult2b1^-/-^*), respectively. Eight weeks later, animals were subjected to middle cerebral artery occlusion; the infarction and neurological scores were assessed at 3 days after stroke. n = 5. **(B)** Fluorescence-activated cell sorting (FACS) was performed to confirm CD45.2 expression 8 weeks after bone marrow transfer. **(C)** Infarct size, representative TTC staining and quantification of infarction. **(D)** Neurological scores. * P < 0.05.

Next, we performed the bone marrow transfer experiments, and the CD45.2 expression was checked using FACS (Figure 4A-B). These recipient mice were subjected to MCAo; 3 days later, the infarction areas were measured by TTC staining. The mice that received bone marrow cells from *Sult2b1^-/-^* mice had a larger infraction size (Figure 4C) and worse neurological scores (Figure 4D) than those that received bone marrow cells from WT mice, suggesting immune cells originating from the bone marrow result in the differences between WT and *Sult2b1^-/-^* mice after ischemia.

### Monocytes are the major contributor to the enlarged infarction of *Sult2b1^-/-^* mice

**Figure 5:**
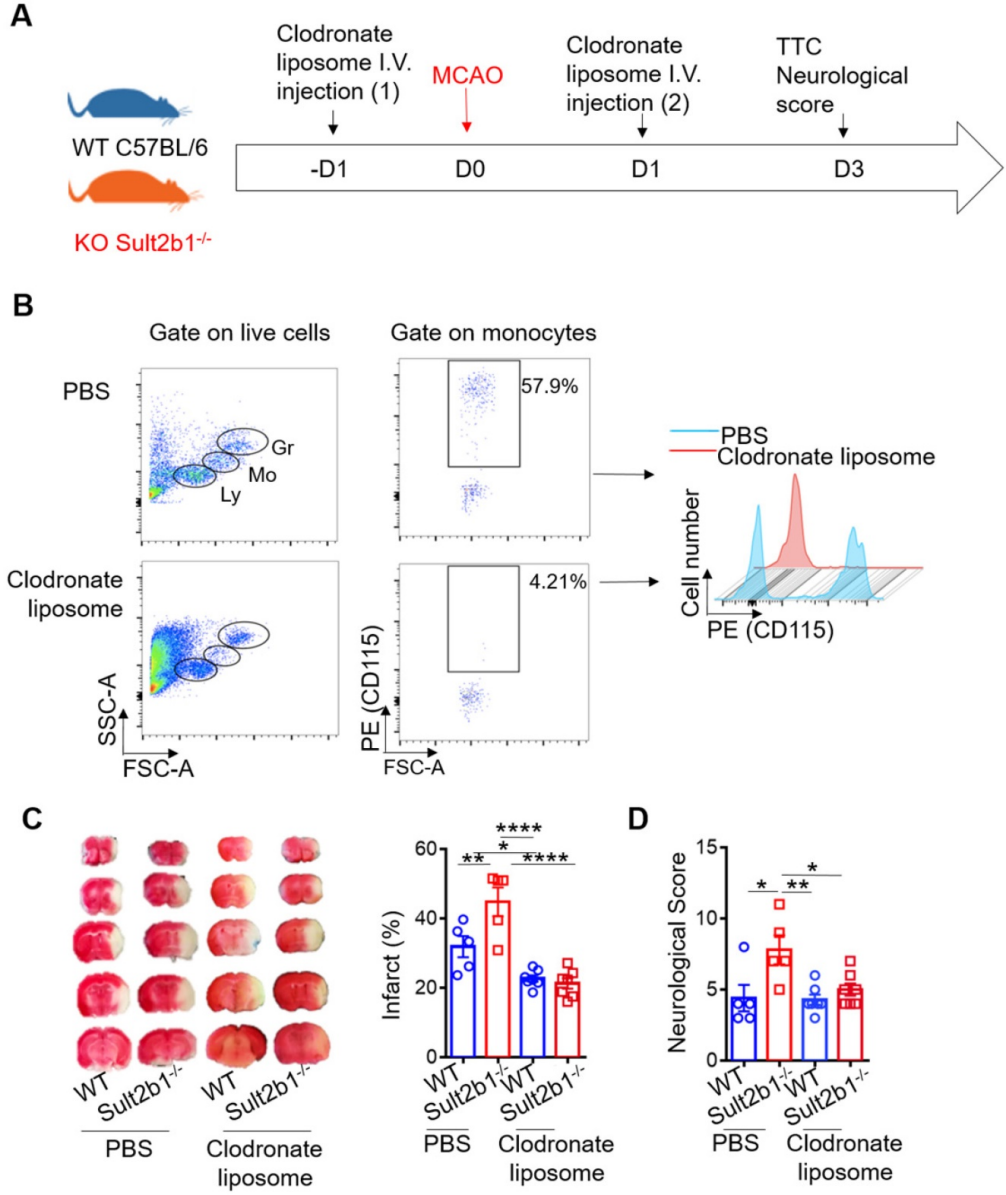
** Circulating monocytes are the major contributor to the enlarged infarction. (A)** Experimental procedure: liposomes were injected 1 day before and 1 day after stroke to deplete monocytes. **(B)** Fluorescence-activated cell sorting (FACS) was performed to confirm monocyte depletion by checking the cell size and granularity and the monocytes cell surface marker CD115. n = 5. **(C)** Representative infarct sizes observed using TTC staining and statistical analysis. **(D)** Neurological scores, * P < 0.05, ** P < 0.01, **** P < 0.0001.

To determine the type of immune cell that plays a major role in determining the size of infarction, we performed the immune cell depletion experiments using clodronate liposome (Figure 5A-B) and anti-Ly6G antibody (Figure S2A-B) to deplete monocytes and neutrophils, separately. While neutrophil depletion did not dramatically change the infarction size (Figure S2), monocyte depletion alleviated the worse stroke outcome in *Sult2b1^-/-^* mice. After monocyte depletion, there was no significant difference between WT and *Sult2b1^-/-^* mice in terms of infarct size and neurological score (Figure 5C-D), suggesting that monocytes may contribute to the worse stroke outcomes in *Sult2b1^-/-^* mice.

**Figure 6:**
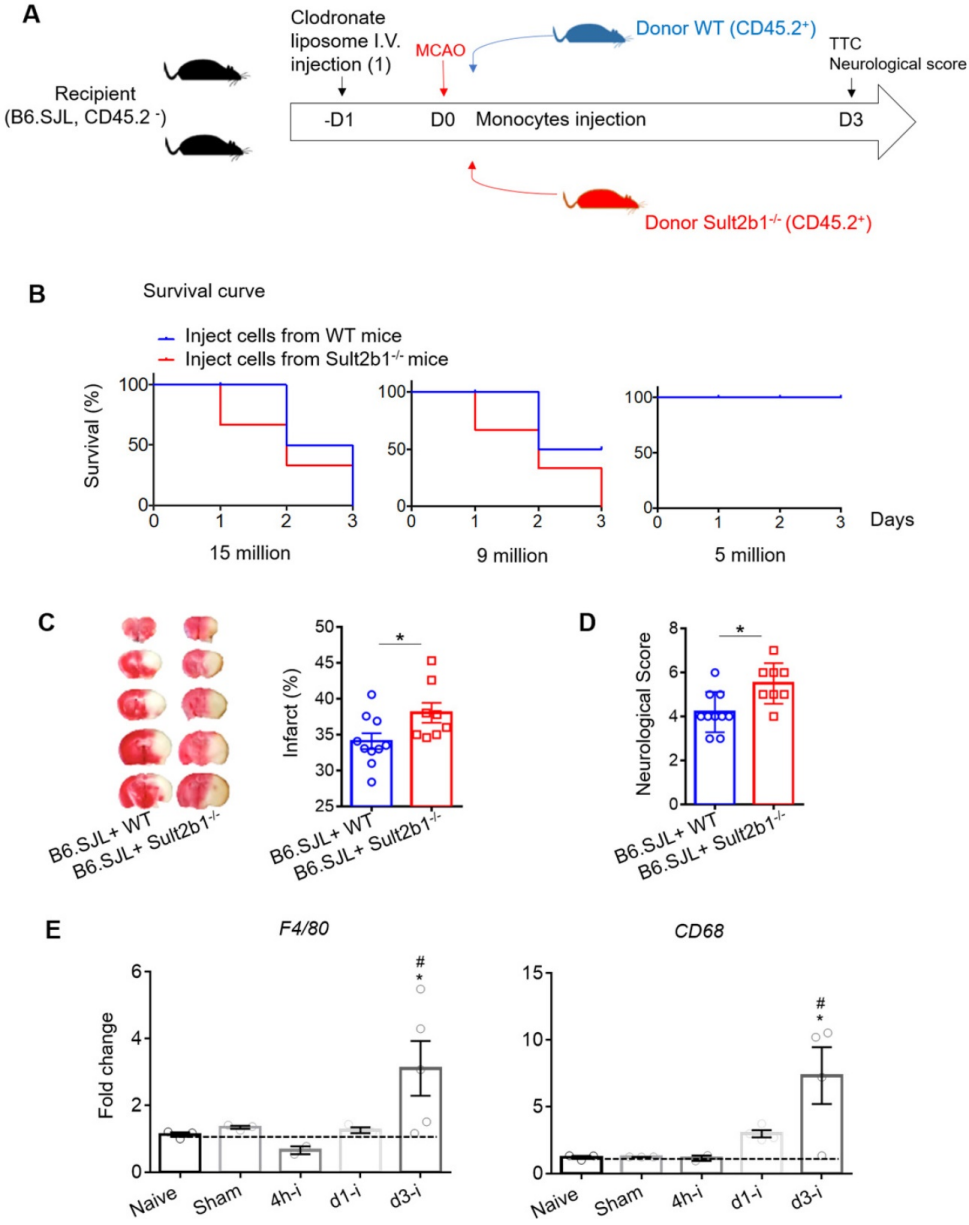
** Adoptive transfer of *Sult2b1^-/-^* monocytes enlarges the brain infarction. (A)** Experimental procedure to study the effects of monocytes transfer on stroke outcomes: the recipient mice were injected with liposome for monocytes depletion. Then, animals were received adoptive transfer of purified monocytes from WT and *Sult2b1^-/-^* mice, n = 10. **(B)** Suitable monocyte cell numbers were selected for i.v. injection by checking mice survival rate. **(C)** Representative infarct sizes using TTC staining and statistical data. **(D)** Neurological scores, * P < 0.05. **(E)** Relative expression of macrophage markers (*F4/80* and *CD68*) in ischemic hemisphere, * vs. naïve, * P < 0.05; # vs. sham, # P < 0.05.

To further confirm the role of monocytes, we performed a monocyte adoptive transfer experiment (Figure 6A). We first performed a cell number transfer test to determine the suitable number of monocytes to transfer back to the WT stroke mice. Our results showed that when 15 million monocytes from WT or *Sult2b1^-/-^* mice were i.v. injected into the ischemic mice, all the mice died within 3 days. When the number decreased to 9 million cells, mice that received monocytes from WT mice had 50% survival rate, while those that received monocytes from *Sult2b1^-/-^* mice all died. When we decreased the injected monocyte numbers to 5 million cells, all mice survived 3 days after MCAo (Figure 6B). Based on these data, we decided to transfer 5 million cells in the subsequent experiments. Next, we confirmed that these i.v. injected CD45.2^+^ cells could last for at least 3 days in the peripheral blood (Figure S3). Using TTC staining, we showed that the recipient mice receiving monocytes from *Sult2b1^-/-^* mice had a larger infarction size and worse neurological scores than those that received monocytes from WT mice (Figure 6C), confirming that monocytes from *Sult2b1^-/-^* mice were indeed play a role in worsening the stroke outcomes.

Furthermore, we purified RNA from the ischemic hemisphere, which showed that 3 days after ischemic stroke, the macrophage markers *F4/80* and *CD68* in the ischemic hemisphere were dramatically elevated compared to the naïve group (Figure 6D) suggesting that macrophages play an essential role in the neuroinflammation induced by ischemia.

### The ischemic hemisphere of *Sult2b1^-/-^* mice accumulates more pro-inflammatory macrophages

**Figure 7:**
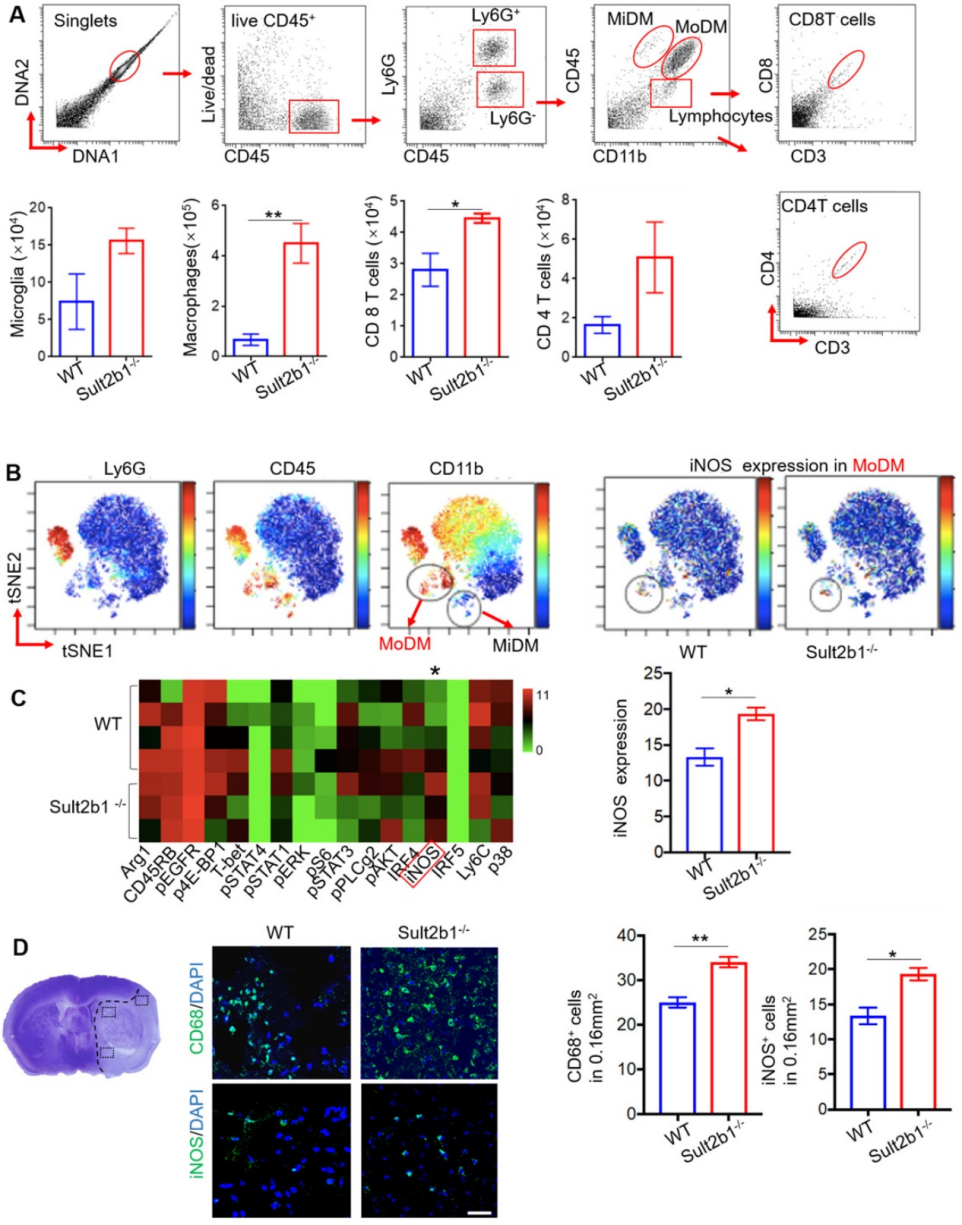
***Sult2b1^-/-^* mice has more pro-inflammatory monocyte-derived macrophages (MoDMs) in the ischemic hemisphere after stroke. (A)** The gating strategy of analyzing the immune cells in the ischemic hemisphere using cytometry by time of flight (CyTOF), and cell numbers of microglia, macrophages, CD4 T cells and CD8 T cells. * P < 0.05, ** P < 0.01. **(B)** ViSNE analysis showed that MoDM (Ly6G^-^, CD45^hi^, CD11b^+^) in *Sult2b1^-/-^* mice expressed higher iNOS levels than WT mice. **(C)** Heatmap showed that the MoDMs in *Sult2b1^-/-^* mice expressed higher iNOS. WT mice, n = 4; *Sult2b1*^-/-^ mice, n = 3. **(D)** Confocal immunofluorescent staining for CD68^+^ and iNOS^+^ cells. The left panel shows where the photos were taken. The middle panel shows the representative staining. The right panel shows the quantification of CD68^+^ and iNOS^+^ positive cells. * P < 0.05, ** P < 0.01.

Using high-dimensional CyTOF techniques, we analyzed the immune cells from the ischemic hemisphere from both WT (n=5) and *Sult2b1^-/-^* (n=3) mice (Figure 7A). Our data showed that, compared to WT mice, the monocyte-derived macrophages (MoDMs) from *Sult2b1^-/-^* mice expressed higher levels of iNOS than those from WT mice (Figure 7B-C). To further confirm these results, we performed the IF staining, which showed that stroke resulted in a significant increase in CD68^+^ and iNOS^+^ cells in *Sult2b1^-/-^* mice (Figure 7D). These data suggested that the increased pro-inflammatory activity of MoDMs results in worsening stroke outcomes in *Sult2b1^-/-^* mice.

### Cholesterol sulfate increases NADPH level, decreases ROS level and activates AMPK-cAMP-CREB signaling pathway

**Figure 8:**
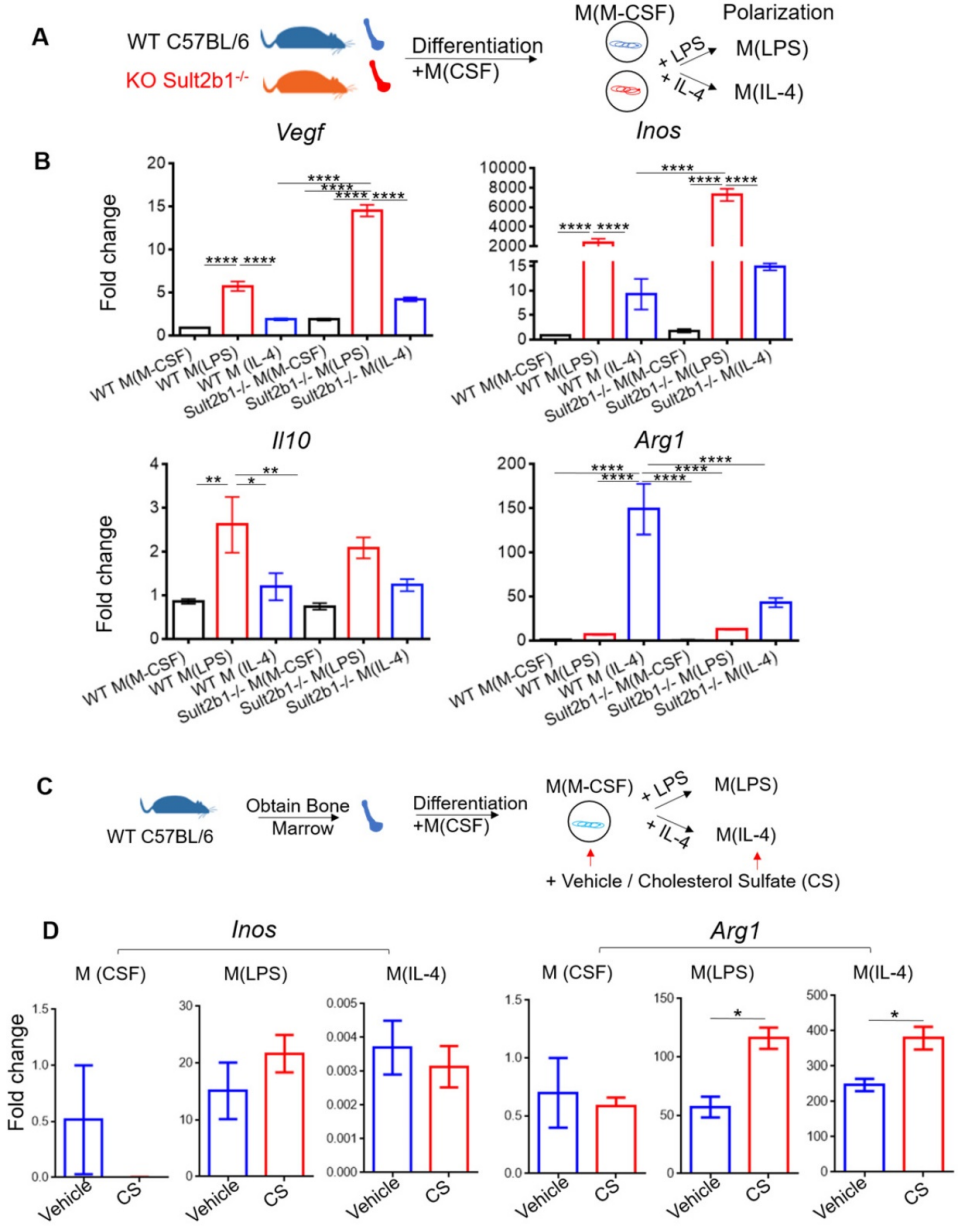
** Cholesterol sulfate attenuates primary macrophage polarized into pro-inflammatory status. (A)** Experimental design and procedure. The bone marrow cells from *Sult2b1^-/-^* mice and WT mice were first differentiated into macrophages and then polarized into pro-inflammatory and anti-inflammatory macrophages. **(B)** Macrophages differentiated from *Sult2b1^-/-^* mice expressed higher pro-inflammatory markers such as *Vegf* and *Inos*, and less anti-inflammatory markers such as *Il-10* and *Arg1*. **(C)** Experimental design and procedure: adding cholesterol sulfate during the differentiation and polarization of monocytes from WT mice. **(D)** The expression of *Arg1* increased, when bone marrow-derived macrophages (BMDMs) were treated with cholesterol sulfate during the polarization process. CS stands for cholesterol sulfate; n = 3 for each group * P < 0.05, ** P < 0.01, *** P < 0.001, **** P < 0.0001.

To investigate the role of *Sult2b1* gene in macrophage polarization, we purified the BMDMs from both WT mice and *Sult2b1^-/-^* mice, and differentiated them into macrophages. The cells were then polarized into M (LPS) and M (IL-4) (Figure 8A). The qRT-PCR data showed that M(LPS) from *Sult2b1^-/-^* mice expressed higher *Inos* and *Vegf* than M(LPS) from WT mice, while for M(IL-4), *Sult2b1^-/-^* mice expressed lower level of *IL-10* and *Arg1* (Figure 8B), suggesting that the *Sult2b1* gene contributes to anti-inflammatory characteristics in macrophages.

Next, we added cholesterol sulfate during the monocyte polarization process (Figure 8C), our data showed that the anti-inflammatory markers (e.g.,* Arg1*) significantly increased (Figure 8D). The addition of 10 µM cholesterol sulfate did not affect the cell viability of primary monocytes (Figure S4A). To provide a broader characterization of the macrophages after adding cholesterol sulfate, we collected the cell supernatants and performed the Luminex assay. The results showed that the anti-inflammatory cytokines, such as IL-10 and IL-4, were significantly increased after adding cholesterol sulfate (Figure S4B), which further confirmed our hypothesis.

**Figure 9:**
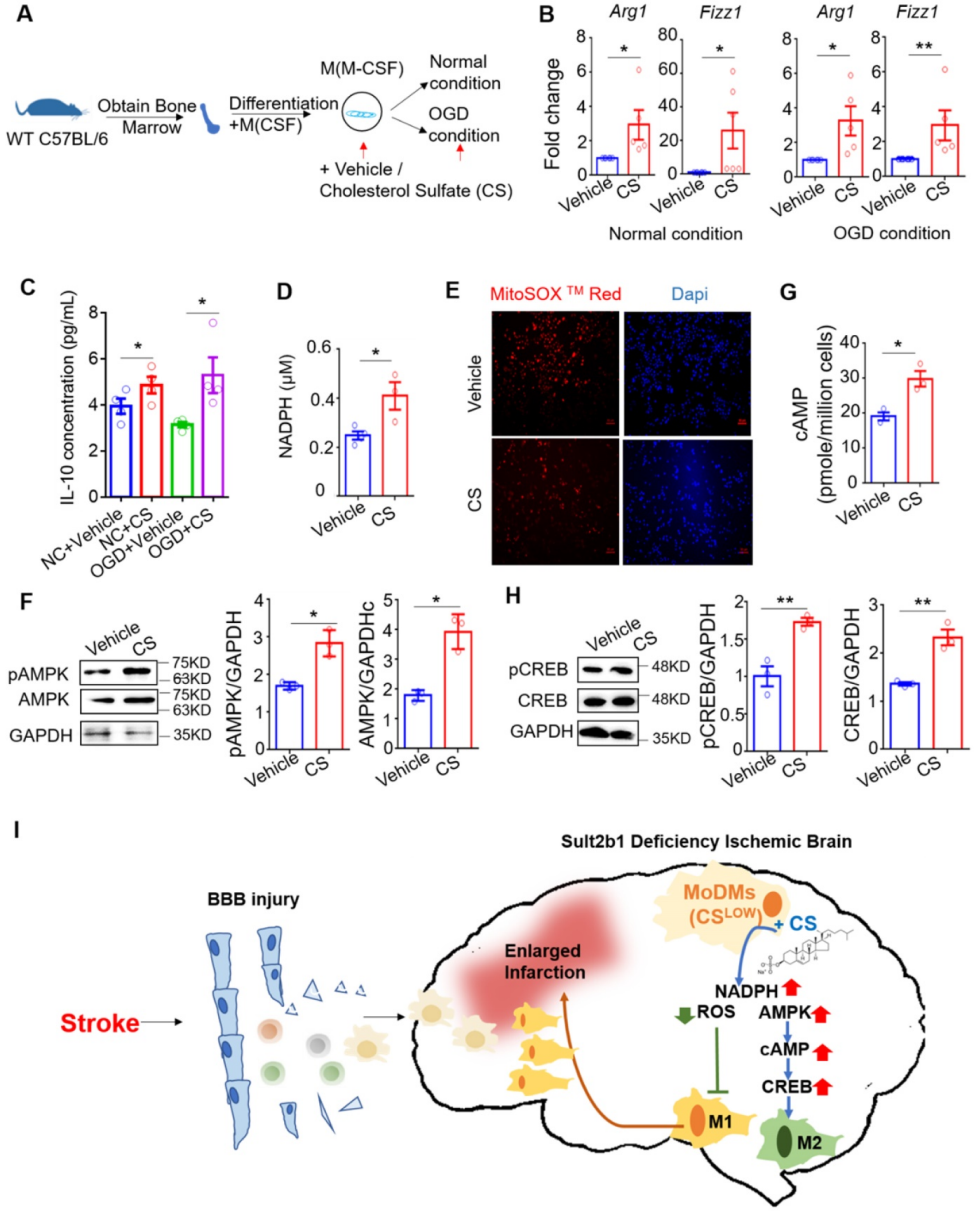
** Cholesterol sulfate attenuates the pro-inflammatory polarization of macrophages. (A)** Experimental design and procedure: primary bone marrow-derived monocytes (BMDMs) were first differentiated into macrophages. Then, the cells were cultured in either normal or OGD conditions with / without cholesterol sulfate treatment. **(B)** RT-PCR results showed that under both normal and OGD conditions, BMDMs treated with cholesterol sulfate had higher expression of *Arg1* and *Fizz1*. * P < 0.05, ** P < 0.01. **(C)** Interleukin-10 (IL-10) ELISA assay showed that under both normal condition and OGD conditions, cells with cholesterol sulfate treatment secreted more IL-10. NC stands for normal condition. **(D)** NAPDH assay showed that the BMDMs treated with cholesterol sulfate had higher NAPDH production. * P < 0.05. **(E)** Fluoresce microscopy showed that the BMDMs treated with cholesterol sulfate produced less ROS. MitoSox in red, Dapi in blue, and the red scale bar on the right bottom stands for 50 µm.** (F)** Western blot showed that the BMDMs treated with cholesterol sulfate expressed higher pAMPK and AMPK. Bar chat shows the density comparison between pAMPK/AMPK vs. GAPDH, * P < 0.05. **(G)** cAMP assay showed that the BMDMs treated with cholesterol sulfate had higher cAMP level. * P < 0.05. **(H)** Western blot showed that the BMDMs treated with cholesterol sulfate expressed higher pCREB and CREB. Bar chat shows the density comparison between REB / CREB vs. GAPDH ** P < 0.01. **(I)** Diagrammatic sketch: upon ischemic stroke, the brain accumulates peripheral immune cells, majorly monocyte-derived macrophages (MoDMs). When *Sult2b1* is knocked out, the macrophages tend to polarize into the pro-inflammatory status, causing a larger infarction size. Treated macrophages with exogenous cholesterol sulfate attenuates this macrophage differentiation into pro-inflammatory status by increasing the NADPH level, decreasing the ROS level, and activating the AMPK-CREB pathway. CS stands for cholesterol sulfate.

After ischemia, the brain microenvironment is under a low-oxygen and low-glucose microenvironment. Oxygen-glucose deprivation (OGD) model is a well-established and commonly used to mimic ischemic stroke *in vitro* 12, 32. The primary BMDMs were cultured under both normal and OGD conditions for 6 h (Figure 9A). The qRT-PCR results showed that, with cholesterol sulfate treatment, the markers of the alternative activation macrophages, *Arg1* and *Fizz1*, were significantly increased compared to the control vehicle group (Figure 9B). We also demonstrated that cells treated with cholesterol sulfate expressed higher levels of IL-10 under both normal and OGD conditions (Figure 9C). This phenomenon was also confirmed using the murine monocytes cell line, raw 264.7 (Figure S5A-D).

We next showed that the cells treated with cholesterol sulfate had higher level of the NADPH (Figure 9D), but lower level of ROS (Figure 9E). We also demonstrated consistent results under OGD conditions using the mouse raw 264.7 cell line (Figure S5E-G). Considering AMPK is a cellular energy sensor molecule, especially during energy stress, we hypothesized that AMPK might be the key signaling linker involved in this process. We then performed western blotting, which demonstrated that BMDMs with cholesterol sulfate treatment had increased the levels of total AMPK and phosphorylated AMPK (Figure 9F). After ischemic stroke, the intracellular cyclic AMP (cAMP) had also been activated 33. Since, stimuli that increase the activity of AMPK have also been shown to increase intracellular cAMP 34, 35. We preformed cAMP ELISA assay, and showed with cholesterol sulfate treatment, the level of cAMP increased (Figure 9G). To further address the signaling pathway, we detected the level of CREB and phosphorylated CREB, as the AMPK-CREB pathway is directly affected by ischemic stroke and closely linked to macrophage polarization 36, 37. The western blotting showed that both CREB and its phosphorylated form were increased with cholesterol sulfate treatment (Figure 9H), suggesting that cholesterol sulfate indeed activated and up-regulated the AMPK-CREB signaling pathway.

In conclusion, our data suggested that *Sult2b1* deficiency lead to the increasing numbers of pro-inflammatory macrophages accumulated in the ischemic hemisphere. Adding exogenous cholesterol sulfate could promote macrophage polarization to an anti-inflammatory status, under both normal and OGD conditions, as cholesterol sulfate increasing the level of NADPH, decreasing the level of ROS and activating the AMPK-CREB pathway (Figure 9I).

## Discussion

In this study, we found that the *Sult2b1*^-/-^ mice had a larger infarctions and worse neurological score than WT mice after MCAo surgery. The enlarged infarction was mainly induced by the peripheral infiltrated MoDMs. This is because in *Sult2b1*^-/-^ mice, more MoDMs migrated to the damaged hemisphere, and these MoDMs were more likely to polarize to a pro-inflammatory status. We further demonstrated that the cholesterol sulfate could relieve ischemia-induced energy depletion by up-regulating NADPH, down-regulating ROS, and activating the AMPK-CREB pathway, suggesting a potential neuroinflammation - metabolism axis after ischemic stroke.

Brain ischemia initiates a serious of events, including accumulation of a great amount of peripheral immune cells into the damaged brain 25 and generation of oxygen free radicals 38. In 2019, Prah et al. showed that cholesterol sulfate played a protective role in brain astrocytes by changing its metabolism to against oxidative stress. It attracted our great interests to investigate the roles of cholesterol sulfate after brain ischemic stroke. As our present study showed that *Sult2b1* deficiency mice induced more BMDMs migrating to the ischemic hemisphere. We were aiming to investigate the metabolic changes in BMDMs after cholesterol sulfate treatment.

NADPH, a metabolic product of pentose phosphate pathway, is a coenzyme and classic molecule involved in many anabolic reactions in cells. One of the important biological function of NADPH is to provide redox power to mediate the cell response to oxidative stress. Our present data showed that after exogenous cholesterol treatment, the level of NADPH in BMDMs were significantly upregulated. This data is consistent with previous studies. Administration of exogenous NADPH was showed significantly protected neurons against ischemia/reperfusion-induced injury, in both mice and rat ischemic stroke models 39. And this protective function not only decreased the infarction in short-term, but also improved the long term mortality and neurological deficit 40, indicating that increased NADPH indeed plays a protective role to the oxidative damage.

However, one concern for the increased NADPH is it might be used as substrates to generate ROS by NADPH oxidases (NOXs). The increased ROS level could overwhelm antioxidant defenses and cause tissue damage after ischemic stroke 41. Furthermore, in macrophages, the mitochondrial ROS controls the LPS-induced pro-inflammatory response 42, and its production facilitates the polarization to pro-inflammatory macrophages 43. Therefore, after showing cholesterol sulfate treatment increased the level of NADPH, we next tested the level of ROS in BMDMs. Interestingly, we showed that with cholesterol sulfate treatment, the level of ROS decreased and BMDMs were promoted polarized to anti-inflammatory status. Our explanation is, firstly, NADPH could inhibit the expression of NOX2 and NOX4, which reduced the chance to use NADPH as a substrate to generate ROS 44. Secondly, increased NADPH level by cholesterol sulfate actives the AMPK pathway. AMPK is a central regulator of cellular metabolism, such as lipid metabolism 45, 46. During energy stress, the upregulated NADPH level is associated with AMPK activation by inhibition of ACC1/2 and increasing fatty-acid oxidation 47. In myocardial ischemic injury, exogenous NADPH activated AMPK pathway, inhibiting the mitochondrial damage and cardiomyocyte apoptosis 48. The NADPH-induced AMPK phosphorylation could be abolished when compound C, an AMPK inhibitor, was added, and cardioprotection role was vanished 48. Pharmacological activation of AMPK could significantly inhibit pro-inflammatory marker, iNOS expression, in monocytes and macrophages 49, 50. Importantly, our data showed that the total level of AMPK and the phosphorylation level of AMPK were both increased with cholesterol sulfate treatment in BMDMs, which is consistent with those previous findings 51.

Furthermore, the reduced cerebral ischemic injury not only accompanied with enhanced phosphorylation of AMPK, but also CREB 37. Considering that the increased cellular cAMP level associated with enhanced CREB, which further promoted the macrophage differentiation into anti-inflammatory status 52, we hypothesized that the protective role of cholesterol sulfate with increased AMPK might active the downstream cellular cAMP level, and its responsive element CREB as well as its phosphorylation form to promote the macrophage differentiate into anti-inflammatory status (Figure 9I).

However, the present study still has some limitations. First, there is a lack of evidence to show cholesterol sulfate can be used as a potential treatment *in vivo* to improve stroke outcomes. This is because careful does evaluation based on both animal research and clinical data is required, as an overdose of cholesterol sulfate may induce platelet adhesion in the peripheral 53. Second, our data were based on young, male mice model, and more work is needed to be done in female and aged mice.

In conclusions, our present study demonstrated that *Sult2b1^-/-^* mice had a larger infarction and poorer behavioral performance due to the accumulation of pro-inflammatory macrophages in the damaged hemisphere. Cholesterol sulfate can not only attenuate the pro-inflammatory polarization of macrophages, but also relieve the OGD induced energy depletion by up-regulating NADPH and down-regulating ROS, indicating its potential use in the treatment of ischemic stroke. This study established an inflammation-metabolism axis of the ischemic stroke through the polarization process of macrophages. Nevertheless, the detailed information that how lipid metabolism affects the energy supply in macrophages under ischemia condition still deserve more attention and investigation.

## Supplementary Material

Supplementary figures and tables.

Supplementary Sult2b1^-/-^ mouse.

Supplementary wild type mouse.

## Abbreviations

Sult2b1: sulfotransferase family 2b member 1
NADPH: nicotinamide adenine dinucleotide phosphate
ROS: reactive oxygen species
DHA: docosahexaenoic acid
WT: wild-type
MCAo: middle cerebral artery occlusion
IF: immunofluorescence
MoDMs: monocytes-derived macrophages
iNOS: inducible nitric oxide synthase
BMDMs: bone marrow derived macrophages
OGD: oxygen-glucose deprivation
PFA: paraformaldehyde
AMPK: AMP-activated protein kinase
cAMP: Cyclic adenosine monophosphate
CREB: cAMP responsive element-binding protein
DAMPs: damage-associated molecular patterns
mNSS: modified neurological severity score
LXR: liver X receptor
KC: kupffer cell
NOX: NADPH oxidase
PPARγ: peroxisome proliferator-activated receptor γ
