# Supplementary material for: Sult2b1 deficiency exacerbates ischemic stroke by promoting pro-inflammatory macrophage polarization in mice
Source: Theranostics. 2021 Nov 1;11(20):10074–90. doi: 10.7150/thno.61646 (PMC8581421; doi:10.7150/thno.61646)
Supplement: Supplementary file 1 — Supplementary figures and tables. [file thnov11p10074s1.pdf]

Supplementary data

Supplementary tables

Table S1 Blood flow records for behavior test mice (confirm successful stroke onset)

**Blood flow (mm/s) after stroke onset surgery**							
WT	No	Ischemic Hemisphere	Non-ischemic Hemisphere	Sult2b1$^{-/-}$	No	Ischemic Hemisphere	Non-ischemic Hemisphere
	1	9	68		1	6	56
	2	11	78		2	21	50
	3	9	60		3	7	93
	4	14	72		4	8	67
	5	5	59		5	2	63
	6	6	68		6	9	79
	7	8	62		7	10	97
	8	6	70		8	6	72
	9	9	67		9	20	75
	10	9	68		10	10	80
	11	5	65		11	6	68

Table S2 Primers used in RT-PCR

**Primer**	**Sequences(5'-3')**
Mouse GADPH Forward	ACCACAGTCCATGCCATCAC
Mouse GADPH Reverse	TCCACCACCCTGTTGCTGTA
Mouse Arg1 Forward	AACACGGCAGTGGCTTTAACC
Mouse Arg1 Reverse	GGTTTTCATGTGGCGCATTC
Mouse Fizze1 Forward	TCCAGCTAACTATCCCTCCACTGT
Mouse Fizze1 Reverse	GGCCCATCTGTTCATAGTCTTGA

Table S3 Antibodies used in CyTOF

**Cell identify markers**		
Antibodies	Clone	Metal lab
Anti-mouse Ly6G	IA8	141Pr
Anti-mouse CD11c	N418	142Nd
Anti-mouse Ly6C	H1.4	162Dy
Anti-mouse CD45	30-F11	147Sm
Anti-mouse CD11b	M1/70	148Nd
Anti-mouse B220	RA3-6B2	176Yb
Anti-mouse CD25	3C7	151Eu
Anti-mouse CD3	145-2C11	165Ho
Anti-mouse F4/80	BM8	159Tb
Anti-mouse CD45RB	C363.16A	145Nd
Anti-mouse CD8	53-6.7	168Er
Anti-mouse TCRβ	H57-597	169Tm
Anti-mouse CD49b	HMa2	170Er
Anti-mouse CD44	IM7	150Nd
Anti-mouse CD4	RM4-5	172Yb
DNA staining		191/193Ir
Live/dead sating		195Pt
Barcoding		102-110Pd
**Phosphorylation markers**		
Anti-mouse p4E-BP1	236B4	149Sm
Anti-mouse pAKT	D9E	152Sm
Anti-mouse pS6	S235/S236	175Lu
Anti-mouse pPLCg2	K86-889.37	144Nd
Anti-mouse pEGFR	D7A5	146Nd
Anti-mouse pStat1	4a	153Eu
Anti-mouse pStat3	4	158Gd
Anti-mouse pStat4	38	174Yb
Anti-mouse p38	T180/Y182	156Gd
Anti-mouse pERK1/2	D13.14.4E	171Yb

Supplementary Figures

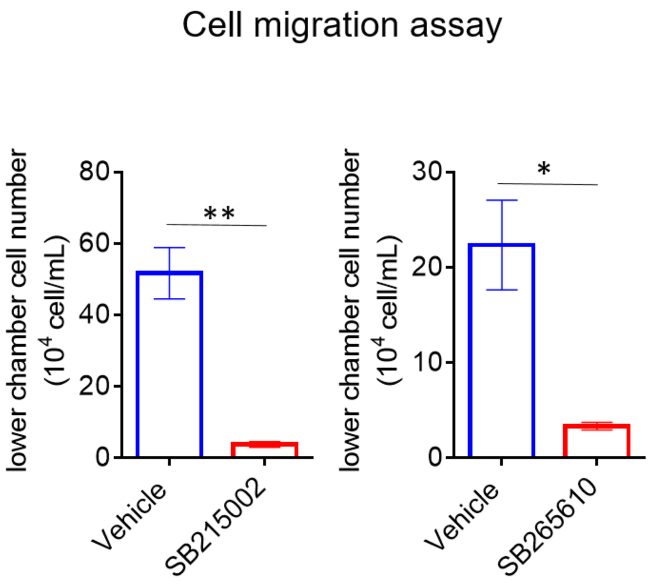

Figure S1: In vitro transwell cell migration assay.

Adding CXCL1 inhibitors (SB215002 or SB265610) decreased the cell migration from the upper chamber to the lower chamber. Vehicle control (cells with dissolve reagent only, without adding any drug).

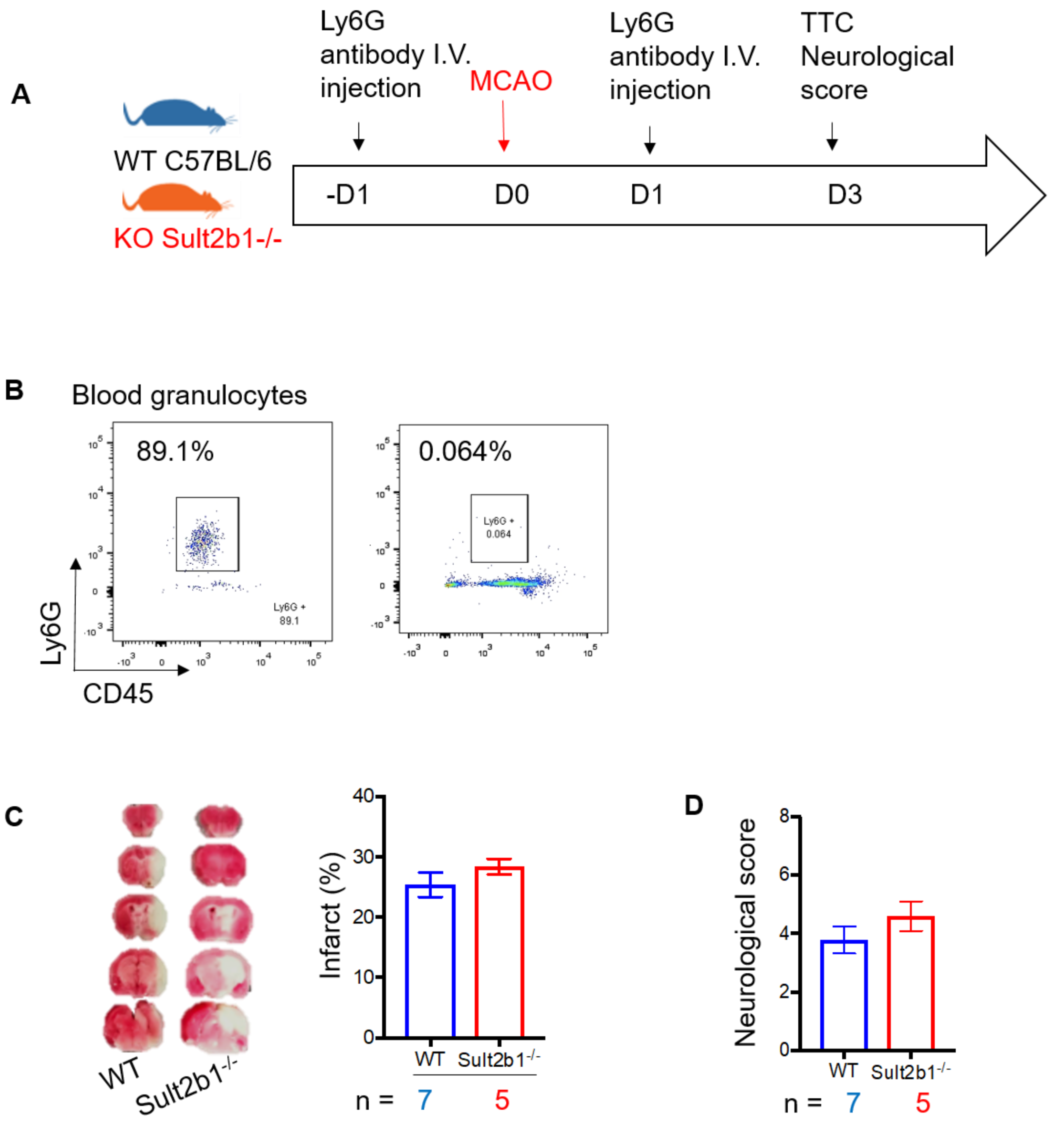

Figure S2: Neutrophils depletion.

For the neutrophil depletion experiment, In vivo Mab anti-mouse Ly6G (clone 1A8) (Bio X Cell, W. Lebanon, NH) antibody was IP injected 1 day before and 1 day after surgery to induce neutrophil depletion (250 µg/mice). The neutrophil population was then monitored using FACS.

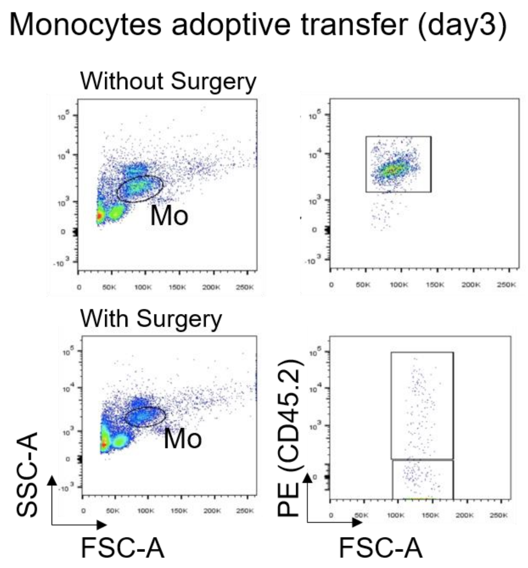

Figure S3: The FACS data to test the CD45.2$^+$ cells in peripheral blood in mice with or without surgery.

Monocytes adoptive transfer B6.SJL-Ptprca Pepcb/ BoyJ (CD45.2$^-$) mice were used as the recipient mice for the adoptive monocytes transfer experiments. Monocytes were first depleted using clodronateliposome via IV injection. After 36h, purified monocytes from Sult2b$^{1-/-}$ mice (CD45.2$^+$) or wild type C57BL/6J mice (Sult2b1$^{+/+}$,CD45.2$^+$) were adoptively transferred via IV injection. The CD45.2 expression was monitored by FACS.

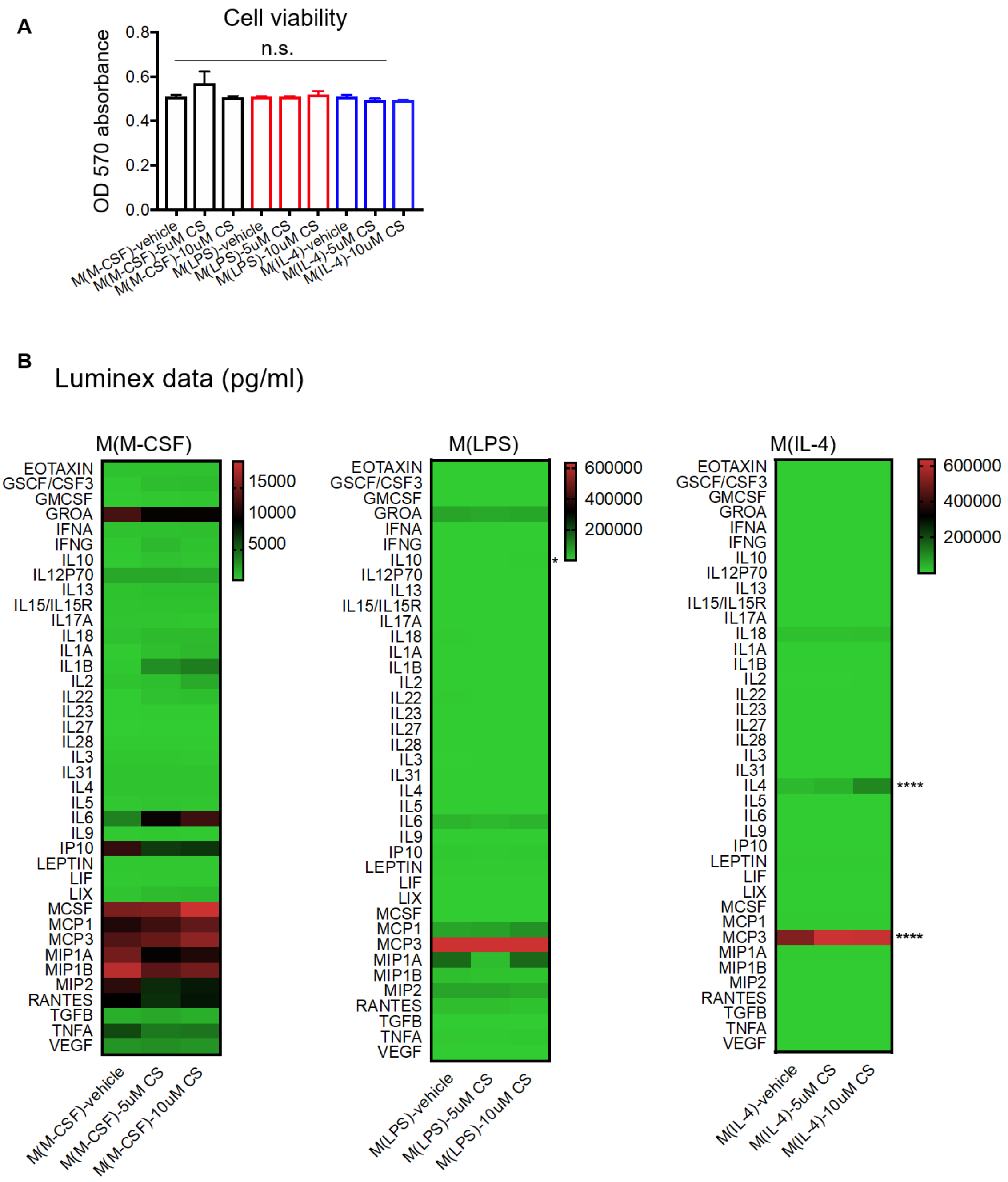

Figure S4：(A) Cell viability assay. First polarized the bone marrow monocytes derived in to macrophages (M-MCF), and then differentiated into pro-inflammatory status (M-LPS) and anti-inflammatory (M-IL4). Adding 5 µM or 10 µM cholesterol sulfate and test the cell viability using alamarBlue™ Cell Viability Reagent (DAL1025, invitrogen) following the instruction. (B) Luminex assay. Collect the cell culture supernatant when polarize the bone marrow derived macrophages, and test the cytokines and chemokines expression level.

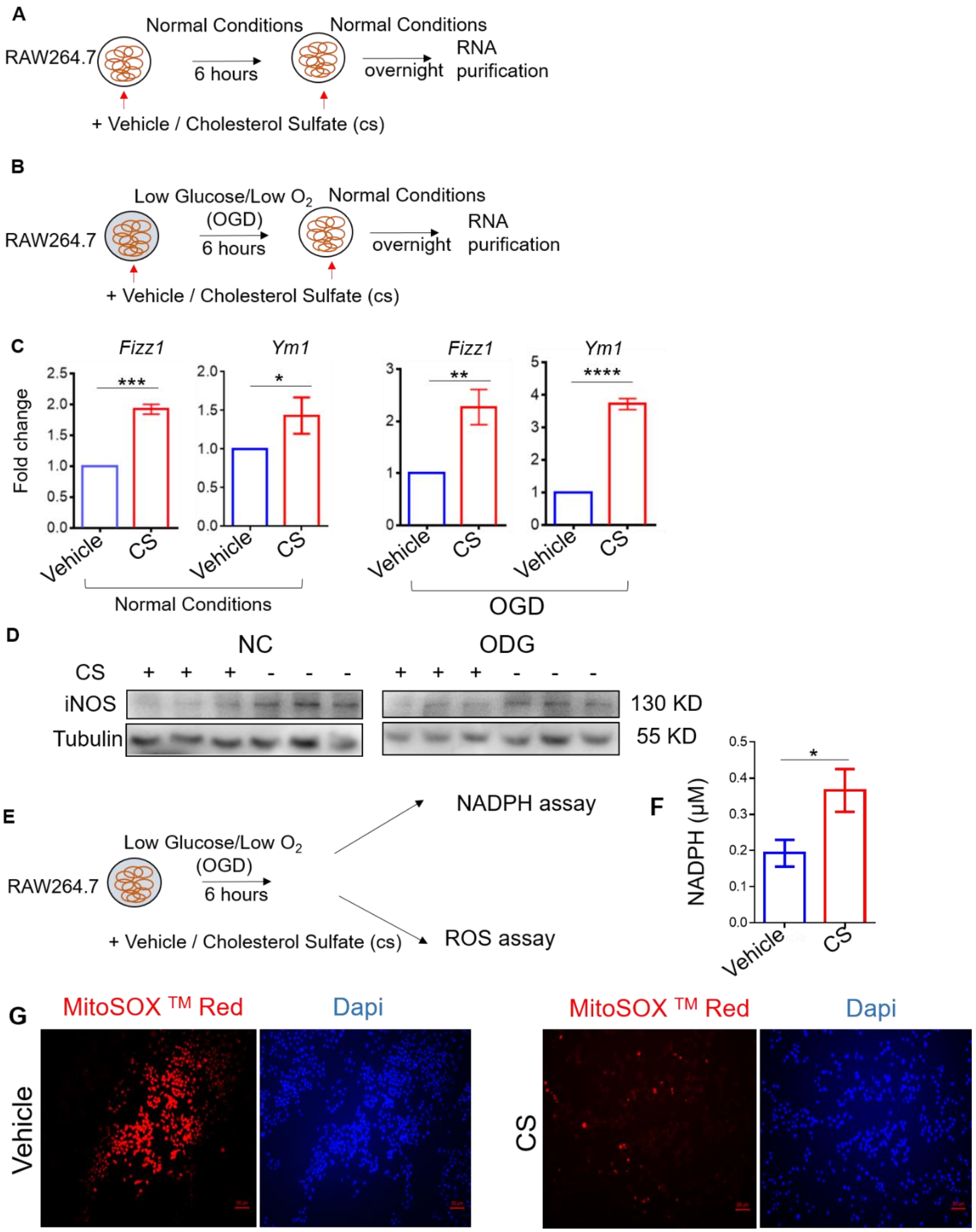

Figure S5 (A-B) The experimental design and procedure. (C) Adding cholesterol sulfate treatment in RAW264.7 increases the expression of anti-inflammation macrophage markers Fizz1 and Ym1 in both normal and OGD conditions. NC stands for normal conditions, OGD stands for oxygen deprivation and glucose deprivation conditions. (D) Western blot shows that with cholesterol sulfate treatment, the iNOS expression significantly decreased compared to control

group in both normal and OGD conditions. (E) The experimental scheme for RAW264.7 NADPH assay and MitoSox test (F) The RAW 264.7 cells were placed in OGD condition for 6 hours, then measured the NADPH concentration and MitoSOX expression. (G) MitoSox in red, Dapi in blue, the write bar stands for 50 μm.

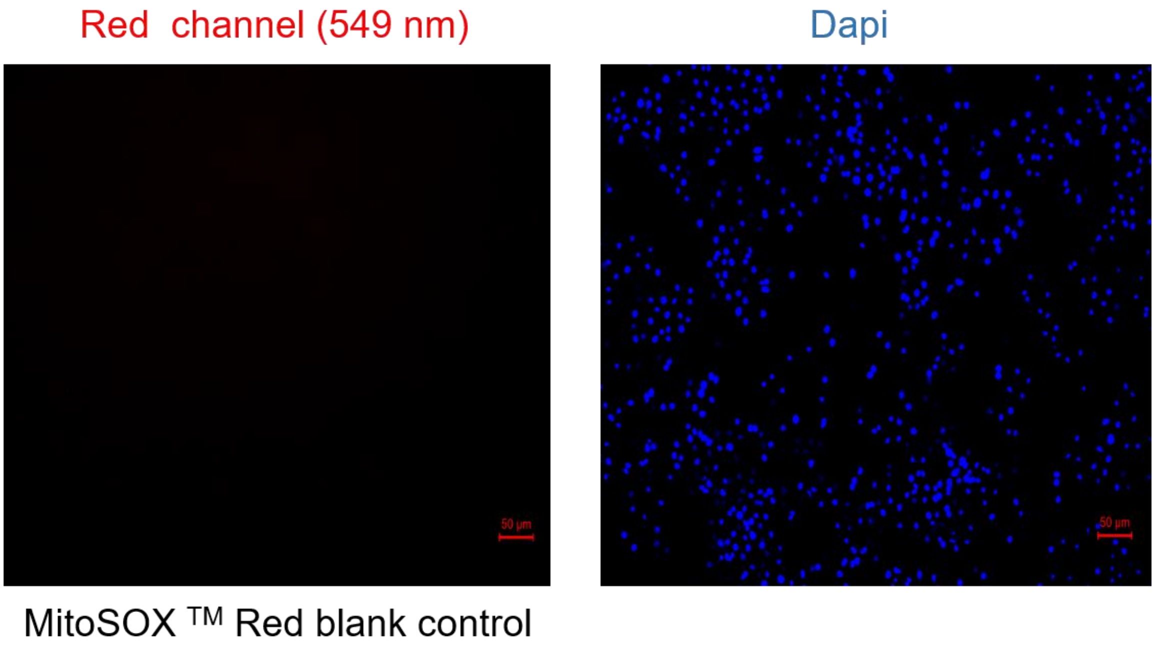

Figure S6: The negative control of MitoSOX Red staining experiment (without adding the Mitosox).
